# Mouse models of metastasis: progress and prospects

**DOI:** 10.1242/dmm.030403

**Published:** 2017-09-01

**Authors:** Laura Gómez-Cuadrado, Natasha Tracey, Ruoyu Ma, Binzhi Qian, Valerie G. Brunton

**Affiliations:** 1 Edinburgh Cancer Research Centre, Institute for Genetics and Molecular Medicine, Edinburgh, EH4 2XR, UK; 2 MRC Centre for Reproductive Health, Queen's Medical Research Institute, University of Edinburgh, Edinburgh, UK; 3 Edinburgh Cancer Research UK Centre, Queen's Medical Research Institute, Edinburgh, EH16 4TJ, UK

**Keywords:** Cancer, Metastasis, Mouse models, Stroma

## Abstract

Metastasis is the spread of cancer cells from a primary tumor to distant sites within the body to establish secondary tumors. Although this is an inefficient process, the consequences are devastating as metastatic disease accounts for >90% of cancer-related deaths. The formation of metastases is the result of a series of events that allow cancer cells to escape from the primary site, survive in the lymphatic system or blood vessels, extravasate and grow at distant sites. The metastatic capacity of a tumor is determined by genetic and epigenetic changes within the cancer cells as well as contributions from cells in the tumor microenvironment. Mouse models have proven to be an important tool for unraveling the complex interactions involved in the metastatic cascade and delineating its many stages. Here, we critically appraise the strengths and weaknesses of the current mouse models and highlight the recent advances that have been made using these models in our understanding of metastasis. We also discuss the use of these models for testing potential therapies and the challenges associated with the translation of these findings into the provision of new and effective treatments for cancer patients.

## Abstract (precis)

**Summary:** This Review describes the currently available mouse models of metastasis, focusing on the mechanistic and therapeutic insights that have been gained by their application, strengths and weaknesses of different models and key technological advances that are generating more refined models.

## Introduction

Metastasis, the process of tumor cell migration from the primary site to distant organs, remains the major cause of cancer-related deaths, despite therapeutic advances in recent years (Steeg, 2016). This highlights the urgent need to better understand the mechanisms that underlie metastasis and to identify new therapeutic strategies and drug targets to treat metastatic disease. A number of excellent review articles have covered the exciting new advances in our understanding of the genetic and molecular events that govern metastatic spread (Lambert et al., 2017; Massagué and Obenauf, 2016; Sethi and Kang, 2011; Turajlic and Swanton, 2016; Valastyan and Weinberg, 2011), which will not be covered in detail here. In this Review, we discuss the different mouse models of metastasis that are currently used, and focus on how they have contributed to the field thus far. We consider their strengths and weaknesses and the technological advances that are driving the development of more refined models, which have the potential to impact on the translation and development of better therapeutic interventions. We first provide an overview of the metastatic process.

## The metastatic cascade

**Fig. 1.**
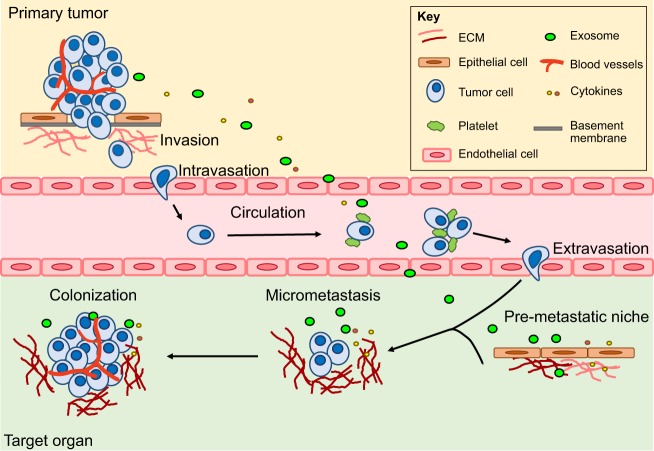
**Metastatic cascade.** Metastasis is a multistep process. Initially, tumor cells migrate into adjacent tissues, referred to as local invasion. This involves breakdown of the basement membrane and invasion into the surrounding ECM. Intravasation then allows cells to enter the circulation. In blood vessels, CTCs exist as single cells or clusters, coated with platelets. They need to survive shear stress and evade clearance by the immune system to successfully reach distant organs. Tumor cells then attach to endothelial cells, which facilitates their extravasation. After settling in the metastatic target organ, tumor cells must survive in this foreign environment and establish micrometastases. These DTCs can remain dormant for many years before proliferating into large macrometastases in a process termed colonization. The primary site also regulates the development of metastasis via secretion of factors (such as cytokines and exosomes) that can prime a pre-metastatic niche (Box 2) and support survival of DTCs. See Glossary in Box 1 for an explanation of key terms.

Metastasis is a multistep process, as illustrated in Fig. 1. The first step of the metastatic cascade is local invasion at the primary tumor site. This process is initiated by the activation of signaling pathways that regulate cytoskeletal dynamics, loss of adhesion amongst tumor cells and turnover of the surrounding extracellular matrix (ECM) (Friedl and Alexander, 2011). This allows the tumor cells to migrate away from the primary tumor and infiltrate into surrounding tissues. To initiate the spread to secondary sites, the tumor cells must then intravasate (see Glossary, Box 1) into the blood circulation or lymphatic system. Dispersal of tumor cells in the lymphatic system leads to lymph node metastasis in the first instance, while distal metastasis usually requires tumor cells to disseminate via the blood circulation (hematogenous) with the choice of a tumor cell to use either lymphatic or hematogenous dissemination, governed by a number of factors (Chiang et al., 2016; Wong and Hynes, 2006). In this article, we will focus on hematogenous metastasis.

**Box 1. Glossary**

**Allograft:** The transplant of cells or organs from one individual to another individual of the same species.

**Cancer-associated fibroblast (CAF):** Fibroblasts found within or in close proximity to a tumor. Usually derived from normal fibroblasts but can also be formed from pericytes (contractile cells that line capillaries) and smooth muscle cells, among other cell types.

**Circulating tumor cell (CTC):** Tumor cells that have left the primary tumor and entered the circulatory system.

**Colonization:** The processes (e.g. survival and proliferation) which allow disseminated tumor cells to form large macrometastases.

**Cre recombinase/*loxP*:** Cre recombinase enzymatically removes sequences that are flanked (floxed) by inserted *loxP* sequences.

**CRISPR/Cas9:** Gene-editing technology that enables precise genomic modifications. This technology can be used to generate gene modifications, deletions and insertions, by using a synthetic guide RNA to introduce a double-strand break at specific sites in DNA, mediated by Cas9 endonuclease.

**Disseminated tumor cell (DTC):** Tumor cells that have settled in distant organs away from the primary tumor site after exiting the circulatory system.

**Ectopic:** The transplantation of a cell type to a location in which it is not found under normal physiological circumstances.

**Epithelial-to-mesenchymal transition (EMT):** Loss of cell-cell adhesion complexes and cell polarity by an epithelial cell, and the gain of an invasive, migratory, mesenchymal phenotype.

**Exosome:** Extracellular vesicles that are released from cells after the fusion of multivesicular bodies with the plasma membrane.

**Extracellular matrix (ECM):** The acellular support surrounding tissues.

**Extravasation:** The process by which a tumor cell leaves the circulatory system and enters a secondary site away from the primary tumor.

**Genetically engineered mouse model (GEMM):** A mouse with a genome altered by genetic engineering techniques, including gene deletion, mutation or addition. This can be performed in a tissue- or cell-specific manner and may also be inducible.

**Immunocompromised mice:** Mice in which specific elements of the immune system have been removed to allow engraftment of human material.

**Integrin:** Transmembrane receptor protein that cells use to adhere and respond to the extracellular matrix.

**Intravasation:** The process by which a tumor cell leaves the primary tumor and enters the circulation.

**Invadopodium:** Actin-rich protrusion present at the membrane of invasive cancer cells that extends and degrades the extracellular matrix.

**Micrometastasis:** Small clusters of cancer cells in secondary organs that are too small to detect through screening.

**Organoids (tumor):** three-dimensional cultures of tumor cells.

**Orthotopic:** The transplantation of a cell type or organ to a location in which it would be found under normal physiological circumstances.

**Patient-derived xenograft (PDX):** A model in which human patient tumor material is implanted into an immunocompromised host, most commonly the mouse.

**Seeding:** In the context of metastasis, seeding refers to the process whereby tumor cells ‘seed’ new tumors in distant organs. Originally described in Stephen Paget's ‘seed and soil’ hypothesis of cancer metastasis (Paget, 1989). The seeding process includes tumor cell adherence to the blood vessel in the distal organ, extravasation, migration to the tissue parenchyma, and survival.

**Syngeneic:** In transplantation biology, this refers to individuals or tissues that are genetically identical or closely related, allowing the transplantation of tissues from the strain of origin into immunocompetent mice.

**Transendothelial migration:** Movement of tumor cells through the endothelial barrier either paracellularly (through the endothelial cell junctions) or transcellularly (through the endothelial cell body).

**Tumor-associated macrophage (TAM):** A macrophage found within or in close proximity to a tumor that actively promotes tumor growth through the secretion of cytokines and chemokines.

**Tumor microenvironment:** All elements that make up the surroundings of the tumor, including other cell types, vasculature and the extracellular matrix.

**Xenograft:** The transplant of cells or organs from one species into an individual of a different species.

**Box 2. Pre-metastatic niche**

Pre-metastatic niches are organ-specific supportive biological environments that support survival of CTCs in distal organs and promote metastatic outgrowth. The formation of the pre-metastatic niche is governed by secreted factors from the primary tumor, including growth factors and inflammatory cytokines and chemokines. Recent studies found that tumor-derived exosomes can also promote niche establishment and determine organ specificity in some types of cancer; however, whether it is a common feature for pre-metastatic niche formation remains unclear. These tumor-derived secreted factors induce changes in distant pre-metastatic sites while also mobilizing bone marrow-derived cells, the recruitment of which – along with a number of other types of immune cell – to the niche leads to remodeling of the local environment and formation of the pre-metastatic niche. This involves interactions with local resident stromal cells, such as endothelial cells, macrophages and fibroblasts, and also the extracellular matrix, which all cooperate to form a permissive environment for tumor outgrowth. For example, activated fibroblasts remodel the ECM by secreting matrix components such as fibronectin and metalloproteinases that break down existing ECM. Increased fibronectin in the niche enhances adhesion of recruited bone marrow-derived cells. Lysyl oxidase, an enzyme that cross-links collagen and elastins in the ECM, is also important in the formation of the pre-metastatic niche; by remodeling the ECM, this enzyme enhances myeloid cell infiltration. For further information, readers are directed to two reviews on the formation and role of the pre-metastatic niche (Liu and Cao, 2016; Peinado et al., 2017).

After entry into the circulation, tumor cells can disseminate widely throughout the body and are known as circulating tumor cells (CTCs) (see Glossary, Box 1). CTCs have the potential to serve as prognostic markers of metastasis and survival, as has been discussed extensively in recent reviews (Alix-Panabierès and Pantel, 2013; Plaks et al., 2013). On reaching distal organs, surviving tumor cells can be intercepted in small capillaries or actively adhere to larger blood vessels and extravasate through paracellular or transcellular transendothelial migration (see Glossary, Box 1) (Reymond et al., 2013), prior to colonization (see Glossary, Box 1). This process can be promoted by alterations induced by secreted factors and extracellular vesicles derived from the primary tumor, before the establishment of metastases (McAllister and Weinberg, 2014). These alterations involve fibroblasts, endothelial cells and immune cells, especially bone marrow-derived immature myeloid cells, which can collectively establish a pre-metastatic niche (Box 2) that provides an environment favoring the recruitment of CTCs and their subsequent growth (Liu and Cao, 2016; Peinado et al., 2017).

Once settled in the metastatic organ, tumor cells are referred to as disseminated tumor cells (DTCs) (see Glossary, Box 1). DTCs can be present for years or decades and stay in a latent state as single cells or micrometastases (Massagué and Obenauf, 2016). This tumor dormancy may result from single DTCs entering a quiescent state or may be due to inadequate vascularization or immune clearance of micrometastases (Gay and Malanchi, 2017). Eventually, clinically relevant macrometastases (see Glossary, Box 1) arise from the outgrowth of DTCs, a process termed colonization (see Glossary, Box 1).

## Models of metastasis

In this section, we provide an overview of the main mouse models of metastatic cancer that are currently in use, from mice generated using transplantable cancer cells and tumors to genetically engineered mouse models (GEMMS) (see Glossary, Box 1) (Francia et al., 2011; Kabeer et al., 2016; Kersten et al., 2016; Saxena and Christofori, 2013). These mouse models are classified and summarized in Table 1, and examples of models that have been used are listed in Table 2.

**Table 1.**
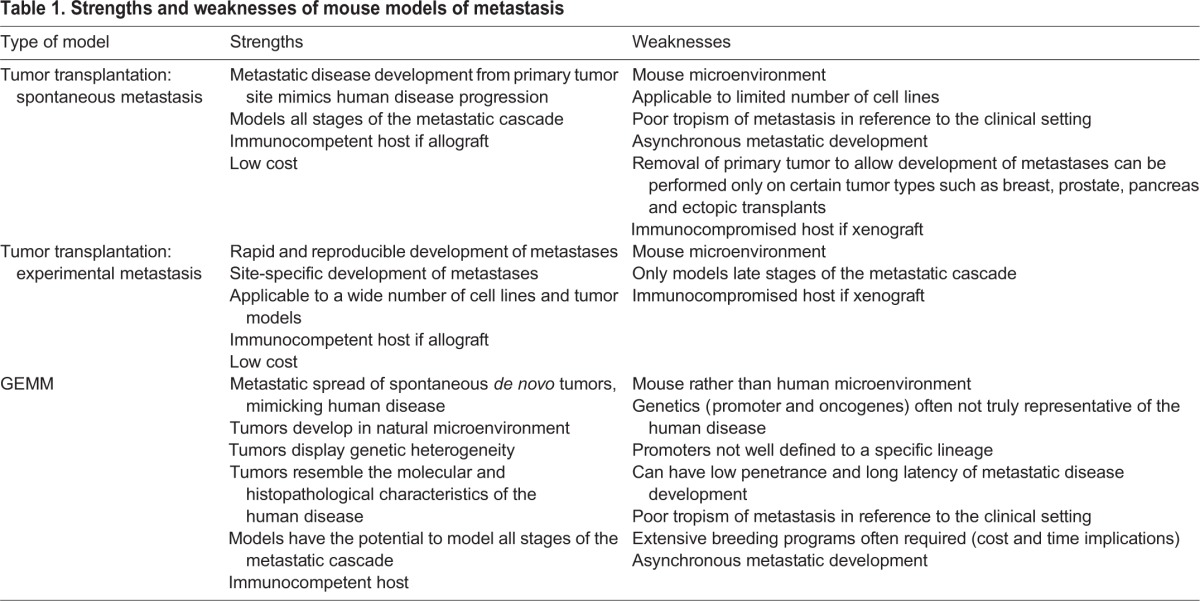
**Strengths and weaknesses of mouse models of metastasis**

**Table 2.**
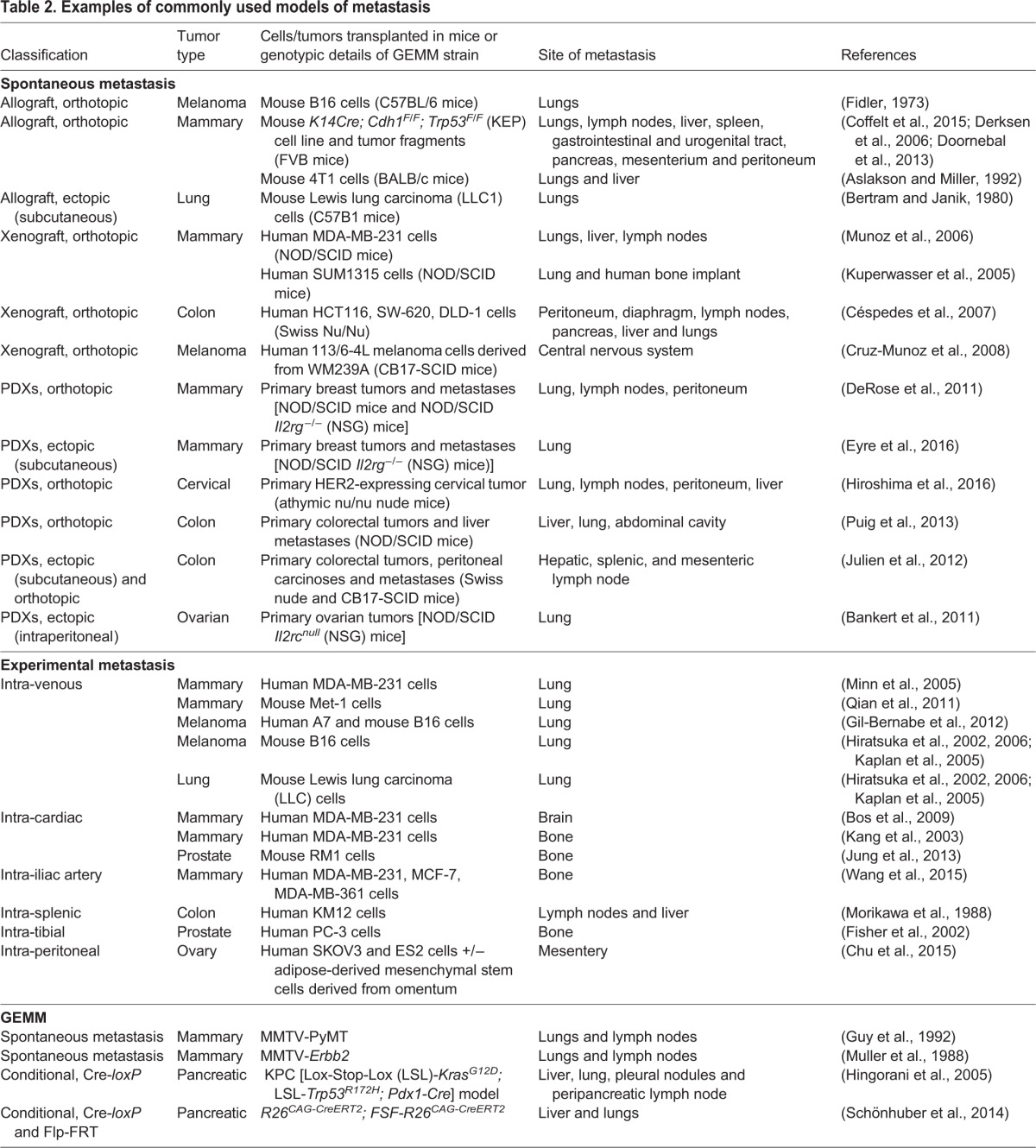
**Examples of commonly used models of metastasis**

### Transplantation models

#### Spontaneous models of metastasis

Spontaneous metastasis models allow the spread of cells from a primary tumor to secondary sites to be followed in animals that have received ectopic or orthotopic (see Glossary, Box 1) transplants of cancer cells or tissue. The advantage of these models is that they allow the entire metastatic cascade to be modeled. However, although ectopic subcutaneous transplantation of multiple cell or tumor types is widely used to monitor tumor growth as it induces rapid tumor growth in the highly vascularized skin, and tumor development can be monitored easily through the use of caliper measurements, metastasis is not often seen in these models and tends to be restricted to allograft models (see below) such as the B16 melanoma and Lewis lung carcinoma model (Table 2). Orthotopic studies are able to better recapitulate human cancers by enabling interactions with the tissue of origin, which can impact on the initial invasion and intravasation and may reflect the increased metastatic spread seen in orthotopic models (Francia et al., 2011; Kellar et al., 2015) (Table 2). This is dependent on the ability to implant tumor cells in the orthotopic site from which the original tumor was derived, which has been successful for a number of tumor types including mammary, pancreatic, lung and colon (Table 2). In some models, there is a long latency, and resection of the primary tumor is needed to allow development of metastases; this is only possible for certain cancers, such as mammary tumors and melanoma (Coffelt et al., 2015; Cruz-Munoz et al., 2008). However, models in which resection of the primary tumor is possible have the benefit of allowing potential adjuvant therapies to be tested.

#### Experimental models of metastasis

Experimental models of metastasis are used to evaluate the capacity of cancer cells to arrest, extravasate and grow in particular organs in ectopic sites following intravascular injection. Different sites of vascular injection in the mouse, including the lateral tail vein, intra-portal, intra-carotid and intra-cardiac, define the site of colonization (reviewed in Khanna and Hunter, 2005). For example, injection into the tail vein leads to the formation of lung metastases, which reflects the rapid trapping of cells within the microvasculature of the lung minutes after injection. Intra-cardiac injection allows wider dissemination of cancer cells and is commonly used to model bone and brain metastasis. Other models include intra-peritoneal injection to model the local dissemination of ovarian cancer or intra-splenic injection of colon cancer cells leading to metastasis formation within the liver. A drawback of experimental metastasis models is that they do not recapitulate the first steps of the metastatic cascade, and only reflect homing of tumor cells circulating in the bloodstream to a limited set of secondary organs. Despite this, they have been instrumental in elucidation of tumor-host interactions required for the initial arrest and colonization at metastatic sites, as discussed below.

Both allograft and xenograft (see Glossary, Box 1) transplantation models are used in spontaneous and experimental metastasis assays and the characteristics of these models are explained in further detail below.

#### Allografts

Allograft transplantation models are generated by the transplantation of mouse-derived cancer cells and tumors into mice. The use of genetically identical syngeneic (see Glossary, Box 1) models, to prevent graft versus host reactions, allows investigation of the immune system in cancer progression and identification of new therapeutic opportunities (Serrels et al., 2015).

Fidler (1973) described the first syngeneic mouse model of metastasis and provided the first demonstration that the metastatic potential of tumor cells could be enhanced through *in vivo* selection. B16 melanoma tumor cells are derived from a spontaneous melanoma that developed in the common C57BL/6 strain of laboratory mice. B16 cells with enhanced metastatic properties were generated after several rounds of *in vivo* selection by subcutaneous injection of melanoma cancer cells into the syngeneic C57BL/6J mouse. The occurrence of metastasis to the lungs increases significantly with the clonally selected tumor lines derived from successive pulmonary metastases (Fidler, 1973).

#### Xenografts

In contrast to allograft models, xenografts involve human cancer cells and tumors. Human tissue must be introduced into immunocompromised (see Glossary, Box 1) or immune-deficient mice in order to prevent rejection by the host. An advantage of these tumor models is that the donor cells are human in origin; however, the key drawback is lack of the host adaptive immune system, which is now recognized to contribute to many aspects of primary tumor growth and metastatic spread (Hanahan and Weinberg, 2011). The use of xenograft mouse models in metastatic studies has been restricted to studies in which highly metastatic variants have been derived through *in vivo* selection (e.g. MDA-MB-231 breast cancer cells, KM12 colon carcinoma cells and WM239A melanoma cells), which overcome the problem of limited metastatic potential (Table 2). This method has also been used extensively to identify gene expression signatures that regulate organ-specific patterns of breast cancer metastasis (Bos et al., 2009; Kang et al., 2003; Minn et al., 2005).

Cancer cell lines often fail to retain the characteristics of the original tumor when cultured *in vitro.* Therefore, they do not reflect the phenotypic and genetic heterogeneity of human cancers and, consequently, xenograft models are poor predictors of clinical responses (Kersten et al., 2016). Patient-derived xenografts (PDXs) (see Glossary, Box 1) have emerged as a potential solution to this problem (reviewed in Whittle et al., 2015). PDXs are generated from resected tumors, propagated directly in immunocompromised mice following orthotopic or subcutaneous injection, avoiding *in vitro* selection pressures. PDXs have been shown to reflect the diversity of human cancer, recapitulating the histology and the metastatic characteristics of the original tumor (DeRose et al., 2011; Eyre et al., 2016; Hiroshima et al., 2016; Julien et al., 2012; Puig et al., 2013). Other studies show that the site and frequency of PDX metastasis may vary from that seen in the patient and the engraftment rate is relatively low. Furthermore, the lack of an intact immune system and the presence of mouse stroma mean that PDXs are not an ideal model for studying the role of the tumor microenvironment in disease progression (Jackson and Thomas, 2017; Pompili et al., 2016; Whittle et al., 2015). To overcome these limitations, humanized xenograft mouse models are being developed, in which the human components of the tumor microenvironment, such as immune cells, peripheral blood and stromal tissue have been engrafted (Bankert et al., 2011; Cassidy et al., 2015; Kuperwasser et al., 2005; Morton et al., 2016). There are also challenges associated with these mice, however, including the technical difficulty of increasing the spectrum of immune cells engrafted while reducing the mouse innate immune response (Shultz et al., 2012). The impact that these humanized mouse models will have on research into metastasis remains unclear at present.

### Genetically engineered mouse models

Genetically engineered mouse models (GEMMs) (see Glossary, Box 1) display *de novo* tumor progression and metastasis formation, usually in an immune-competent tumor microenvironment. This enables both the tumor cell-autonomous and stromal influences on all stages of the metastatic cascade to be modeled, making GEMMs an invaluable resource for studying metastasis. Their use in cancer research has been widely reviewed (Kabeer et al., 2016; Kersten et al., 2016; Saxena and Christofori, 2013). Here, we provide an overview of the models available and discuss their potential for modeling metastatic disease.

The first transgenic models used tissue-specific promoters, such as the mammary-specific MMTV promoter, to drive expression of oncogenes such as *v-ErbB2* and *v-HRas* (Muller et al., 1988; Sinn et al., 1987). This was followed by the generation of tumor suppressor gene knockout mice (e.g. *Trp53*) that have a predisposition to tumor formation (Donehower et al., 1992). Although these models have given insight into many fundamental aspects of cancer biology, it is difficult to model sporadic cancer development seen in humans due to lack of tissue specificity of gene knockouts and control of transgene expression in specific cell lineages (Kersten et al., 2016).

More advanced models allow conditional activation of oncogenes and/or inactivation of tumor suppressor genes in somatic cells. The Cre recombinase/*loxP* system (see Glossary, Box 1) is used widely for this. Using this system, genes that are flanked by *loxP* recombination sites are deleted following activation of Cre recombinase (Gu et al., 1993). Use of tissue-specific promoters to drive Cre recombinase expression, combined with expression of oncogenes that are known to be associated with development of human tumors in those tissues, has resulted in the generation of models that recapitulate many of the molecular characteristics and histopathological features of the human disease (Hingorani et al., 2003; Holen et al., 2017; Jackson et al., 2001; Shibata et al., 1997). These have been invaluable in dissecting the complexities of human cancer and its progression.

Further control can be achieved by using regulatable systems such as the Cre-ER system, in which the hormone-binding domain of the estrogen receptor (ER) is fused to Cre recombinase: treatment of mice with the estrogen analogue tamoxifen leads to activation of Cre recombinase in a temporal manner (Lewandoski, 2001). The tetracycline-inducible system also permits the switching on or off (Tet-On/Tet-Off system) of a specific gene of interest in a tissue- and time-specific manner following administration of doxycyline (Gunther et al., 2002). This temporal and spatial control of gene activation and inactivation is useful for overcoming unwanted effects that could impact on organ-specific development or result in embryonic lethality, as seen upon deletion of the *Rb1* tumor suppressor (Lee et al., 1992). These models can also be used to address the importance of genetic changes at specific times during tumor progression.

An important limitation of GEMMs is the low incidence of metastatic spread that often does not reflect the organ tropism seen in the human disease (Kabeer et al., 2016; Kersten et al., 2016). Although identification of the most appropriate tissue-specific promoters and the correct combination of genetic alterations has provided more representative models (Derksen et al., 2006; Hingorani et al., 2003), tumor spread to some metastatic sites has been more difficult to model. For example, it has been challenging to model the development of bone metastases, a common site of secondary lesions in prostate and breast cancer (Rampetsreiter et al., 2011). A second drawback of GEMMs is that in many cases the long latency requires mice to be sacrificed due to primary tumor burden before metastatic lesions have developed. This can be overcome by removal of the primary tumor, after which the subsequent development of metastases can be monitored (Coffelt et al., 2015; Doornebal et al., 2013).

## Applications of mouse models in metastatic research

Despite the limitations discussed above, mouse models have made important contributions to our understanding of cancer progression and metastasis. Here, we highlight some recent advances in which mouse models have been instrumental in defining the key drivers and features of metastatic cancer. We also outline the usefulness of these models as preclinical drug development tools.

## Stromal cell interactions at the primary tumor site

**Fig. 2.**
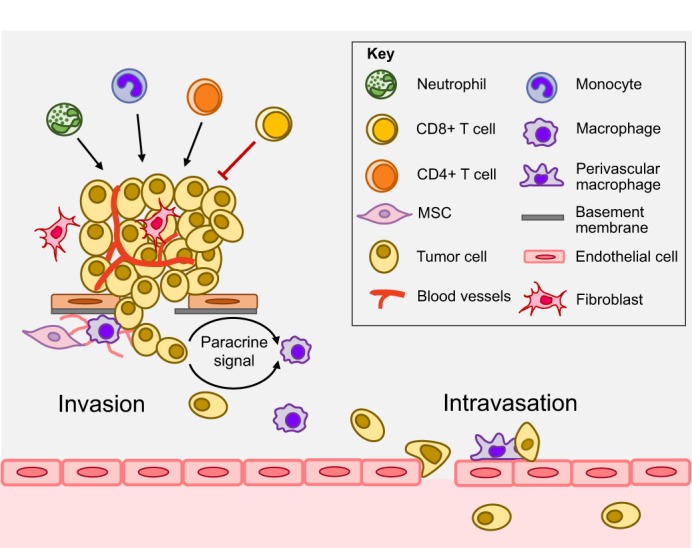
**Stromal influences in the primary tumor.** Stromal cells such as MSCs, fibroblasts and myeloid cells (including monocytes, macrophages and neutrophils) promote metastasis and modulate the tumor microenvironment. MSCs remodel the ECM and support invasion. Macrophages can promote tumor invasion via several paracrine signaling factors. For example, in response to tumor cell-derived CSF1, TAMs secrete EGF, which is permissive for tumor cell invasion and migration. A subset of CD4^+^ T cells contributes to tumor progression, while CD8^+^ T cells mainly mediate anti-tumor immune responses. During intravasation, perivascular macrophages interact with tumor cells directly to help subsequent tumor cells to transit the endothelial barrier and initiate the journey of metastatic dissemination. See Glossary in Box 1 for an explanation of key terms.

*In vivo* metastasis models are important tools to investigate the interaction of tumor cells with tumor-associated stromal components. Within this complex microenvironment, several immune cell populations have been shown to promote tumor invasion and metastasis (Fig. 2) and GEMMs and syngeneic models, both of which have intact immune systems, have provided many valuable insights into how the immune system regulates metastatic progression. Macrophages are often the most abundant infiltrating immune cells in the tumor. These tumor-associated macrophages (TAMs) (see Glossary, Box 1) play multiple roles in promoting cancer metastasis and are associated with metastasis and poor prognosis (Kitamura et al., 2015; Qian and Pollard, 2010). Intra-vital imaging studies have proven useful in elucidating the complex role of TAMs. In a spontaneous GEMM of mammary cancer, MMTV-PyMT (Table 2), cancer cells invade surrounding tissues together with TAMs (Wyckoff, 2004). In this process, TAMs secrete epidermal growth factor (EGF) to activate the EGF receptor on cancer cells, and enhance their motility and invasive potential by increasing invadopodium formation and ECM degradation (Zhou et al., 2014). Reciprocally, tumor cells produce colony-stimulating factor 1 (CSF1) to recruit and activate TAMs. Ablation of this paracrine signal loop significantly inhibited tumor cell invasion in MMTV-PyMT tumors and subsequent lung metastasis without affecting primary tumor growth (Wang et al., 2009). Intra-vital imaging of MMTV-PyMT tumors has also illustrated that tumor cell intravasation occurs in association with perivascular TAMs (Wyckoff et al., 2007). Via direct interactions, invasive tumor cells, perivascular macrophages and endothelial cells form micro-anatomic structures within the tumor (termed ‘tumor microenvironment for metastasis’ or ‘TMEM’) (Pignatelli et al., 2014). The frequency with which these TMEMs occur has been shown to predicate metastasis of ER^+^ breast cancer in a case-control study with >3700 patient samples (Rohan et al., 2014). These TMEM structures control tumor cell intravasation by transiently increasing local vascular permeability in a vascular endothelial growth factor (VEGF)-dependent manner, as illustrated by recent studies using high-resolution two-photon intra-vital microscopy (Harney et al., 2015). These data strongly indicate that the local tumor microenvironment and macrophage interactions play a central role in promotion of tumor cell intravasation, and highlight the importance of using intra-vital microscopy to visualize these processes.

Neutrophils have also been shown to contribute to invasion and metastasis. Using a combination of GEMM and transplant models of melanoma, UV radiation was shown to promote neutrophil infiltration through secretion of high-mobility group protein B1 (HMGB1) derived from UV-damaged keratinocytes. This neutrophil recruitment led to enhanced tumor migration and invasion resulting in distal metastasis (Bald et al., 2014). More recently, a role for neutrophils in the pre-metastatic niche within the lung was established using the MMTV-PyMT model (Wculek and Malanchi, 2015). This was mediated through secretion of leukotrienes from neutrophils in the pre-metastatic niche, which promoted metastatic formation. In the KPC (Lox-Stop-Lox (LSL)-*Kras^G12D^;* LSL-*Trp53^R172H^; Pdx1-Cre*) GEMM of pancreatic cancer, where mutations in *Kras* and *Trp53* drive the development of spontaneous pancreatic ductal adenocarcinomas (Gopinathan et al., 2015), neutrophils have also been shown to promote metastasis (Steele et al., 2016)*.*

Among adaptive immune cells, Th2 cells, CD4^+^ T helper cells expressing Type 2 cytokines [e.g. interleukin (IL) 4, IL10], have been shown to promote tumor progression via activation of humoral immunity and inflammation (Shurin et al., 1999). Using the MMTV-PyMT model, it was found that CD4^+^ T helper cells can induce alternative activation of TAMs and secretion of EGF, to directly promote tumor invasion and egress from the primary tumor as a result of IL4 activation (DeNardo et al., 2009). By utilizing existing and emerging mouse models, future experiments could uncover the role of other lymphocyte populations in tumor progression and invasion.

Coffelt et al. (2015) used a GEMM of invasive lobular breast cancer, the *K14Cre; Cdh1^F/F^; Trp53^F/F^* (KEP) model, and transplanted tumor fragments from this KEP model into recipient mice. Metastatic development was monitored following resection of the primary tumor, and the authors observed that systemic expansion of neutrophils significantly promoted spontaneous metastasis to the lungs and lymph nodes by suppressing a CD8^+^ T cell-mediated anti-tumor immune response (Coffelt et al., 2015). Mechanistically, this involves IL17 expression from γδ T cells that leads to expansion of neutrophils via granulocyte colony stimulating factor (G-CSF). IL17 derived from a subset of CD4^+^ T helper cells has also been shown to promote anti-tumor immune responses through the recruitment of dendritic cells and cytotoxic cells in several murine tumor models (reviewed in Zou and Restifo, 2010). Thus, the role of IL17 in metastasis may be dependent on the cancer type and/or specific tissue environment.

Cells of mesenchymal origin, most notably mesenchymal stem cells (MSCs) and cancer-associated fibroblasts (CAFs) (see Glossary, Box 1) can also promote metastasis through direct interaction with tumor cells. For example, MSCs have been shown to promote peritoneal dissemination of ovarian cancer cells via activation of the matrix metalloproteinases (MMPs) MMP2 and MMP9 (Chu et al., 2015). In xenograft and allograft models of prostate cancer, chemokine (C-X-C motif) ligand 16 (CXCL16)/CXC receptor 6 (CXCR6) chemokine signaling promotes recruitment of bone marrow-derived MSCs and their differentiation into CAFs, which in turn promote prostate tumor cell invasion and metastasis through production of chemokines such as CXCL12 (Jung et al., 2013; Mognetti et al., 2013) and chemokine (C-C motif) ligand 5 (CCL5) (Luo et al., 2015). CAF-derived MMPs can promote ECM remodeling and tumor cell invasion (Kalluri and Zeisberg, 2006), and in an orthotopic model of colon cancer, CAFs promote formation of distal metastases through secretion of the glycoprotein stanniocalcin 1 (STC1) which regulates intravasation of the tumor cells (Pena et al., 2013). CAFs can also generate mechanical pressure and paracrine signaling to promote tumor invasion and metastasis (Karagiannis et al., 2012), and through remodeling of the ECM (Kaushik et al., 2016).

In summary, *in vivo* metastasis models have provided important insights into the interactions of tumor and stromal cells that contribute significantly to tumor invasion and metastasis. Of note, only a minority of cancer cells are migratory, as revealed by intra-vital imaging studies across multiple tumor models, even in aggressive tumors (Condeelis et al., 2005; Scheele et al., 2016). Thus, specific interactions of the tumor microenvironment with these migratory tumor cells could be attractive targets to treat metastatic disease.

## Systemic influence on metastasis

The metastatic process is not only influenced by cell-cell interactions within the adjacent primary tumor microenvironment but also systemic alterations induced by the presence of tumor cells. Experimental metastasis assays in tumor-bearing animals have been key in demonstrating that primary tumor-derived systemic factors, such as cytokines and immune cell chemoattractants, can alter metastatic target tissues and influence the subsequent seeding (see Glossary, Box 1) of tumor cells in these tissues (Hiratsuka et al., 2002, 2006; Kaplan et al., 2005). More recently, elegant mouse experiments again using experimental metastasis models have shown that tumor-derived exosomes (see Glossary, Box 1) induce pro-metastatic progenitor cells in the bone marrow through receptor tyrosine kinase MET signaling (Peinado et al., 2012), and that exosomal integrins (see Glossary, Box 1) can direct organ-specific colonization by priming the metastatic niche (Box 2) (Hoshino et al., 2015). Such advances will impact on how we can harness the ability to measure specific systemic factors in clinical samples to allow more careful monitoring of tumor progression.

Systemic influences can also promote colonization after metastatic seeding. For example, a study using an esophageal cancer model showed that lung metastatic colonization (following tail vein injection of tumor cells) can be significantly promoted by distal tumors in an insulin growth factor (IGF)-II-dependent manner (Li et al., 2014). Using a xenograft model in which human breast cancer cell lines with different tumorigenic potential were injected contralaterally into the same mouse, pro-angiogenic cytokines secreted by human luminal breast cancer cells have been shown to mobilize pro-angiogenic vascular endothelial growth factor receptor 2 (VEGFR2)^+^ bone marrow-derived cells into distal tumors to promote angiogenesis (Kuznetsov et al., 2012). However, systemic influences can also be anti-metastatic. Using human prostate and breast cancer cells, and a combination of spontaneous and experimental metastasis models, an earlier study suggested that the presence of a primary tumor can inhibit metastatic seeding. This was mediated through secretion of prosaposin (a precursor of saposins, which function as cofactors for sphingolipid hydrolases), which stimulates expression of the angiogenic factor thrombospondin-1 in lung stromal cells (Kang et al., 2009). Thus, models that faithfully mimic systemic influences in patients are required to better understand the influence of secreted factors on metastasis.

Immune rejection is a key factor that limits the efficiency of tumor engraftment in immune-competent preclinical models, even when the tumor and host are both from the same syngeneic background (Dunn et al., 2006). It is probably not surprising that immune suppression generated by established tumors enhances engraftment efficiency of subsequent (secondary) tumors (Mullen et al., 1985; Reilly et al., 2000). Careful experiment design is essential to study tumor-tumor and tumor-host interaction in these models.

## Role of epithelial-to-mesenchymal transition in metastasis

Epithelial-to-mesenchymal transition (EMT) (see Glossary, Box 1) is a developmental program that occurs during embryogenesis (Thiery et al., 2009). It involves the loss of cell-cell adhesions, apical-basal polarity and the conversion to a mesenchymal phenotype that is typified by increased motility and invasiveness and plays a role in the invasion of tumor cells, an early event in the metastatic cascade (Valastyan and Weinberg, 2011). It is tightly controlled by a number of pathways that activate the EMT transcription factors Snail, Slug, Twist, Zeb1 and Zeb2 (Thiery et al., 2009). However, there is debate concerning the extent to which EMT contributes to the different stages of the metastatic cascade, much of which has arisen from difficulties in demonstrating that mesenchymal cells persist in metastatic lesions (Lambert et al., 2017; Yeung and Yang, 2017). This is confounded by the inherent plasticity exhibited by tumor cells and the reversibility of the EMT program. Since the use of Cre recombinase technology has become more prevalent, the opportunity to study EMT in mouse models and address these issues has advanced greatly. Prior to this, GEMMs to address the causal connection between EMT and metastasis implicated in *in vitro* studies were lacking.

**Fig. 3.**
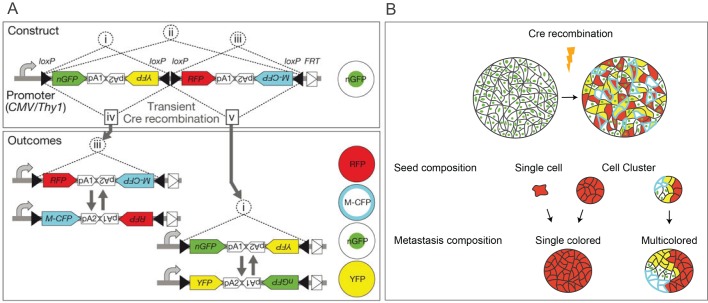
**Lineage tracing allows identification of the clonal nature of metastatic lesions.** (A) The *Brainbow-2.1* construct contains two tandem invertible DNA segments, each flanked by *loxP* sequences (indicated with black arrowheads). Inversion (i-iii) and excision (iv,v) recombination events create four expression possibilities, with the fluorescent protein that follows the promoter being uniquely expressed. Expression of the different fluorescent proteins at different ratios within each cell provides a unique color combination for each cell. Adapted from Livet et al. (2007), with permission from Macmillan Publishers Ltd. (B) Cre-mediated recombination during primary tumorigenesis allows identification of metastatic lesions of a single color, indicating derivation from single cells or clusters from a single cell, or multicolored cell clusters consisting of many different colored cells.

Utilizing Cre recombinase to perform lineage-tracing experiments has provided useful insights into the contribution of EMT to the metastatic process (Fig. 3). One such study involved the widely used KPC model. In these mice, EMT was identified in premalignant lesions, and the process was found to be associated with invasion of the surrounding basement membrane (Rhim et al., 2012). In addition, inflammation enhanced EMT and entry of tumor cells into the circulation. However, using the same KPC model, direct involvement of EMT in the metastatic process was not supported; conditional deletion of *Snai1* or *Twist1*, the genes that encode Snail and Twist, respectively, in the primary tumor resulted in a reduced number of cells undergoing EMT, but this had no impact on the metastatic spread (Zheng et al., 2015). More recently, it was shown that loss of Zeb1 in the KPC model is sufficient to significantly reduce metastatic spread (Krebs et al., 2017). This highlights the specificity and lack of redundancy between EMT transcription factors in controlling metastatic spread in this model.

Using mammary tumor models driven by MMTV-PyMT or human epidermal growth factor receptor 2 (HER2) oncogenes, Fischer and colleagues also established that EMT and metastasis might not be as intricately linked as first thought (Fischer et al., 2015). By using a mesenchymal-specific (Fsp1) promoter to drive Cre recombinase-dependent expression of a green fluorescent protein (GFP) reporter, they observed no enrichment of tumor cells expressing GFP in the metastatic site, and thus no indication of an EMT, although there was an enrichment of GFP-expressing CTCs. A key limitation of this study is the reliance on a single ‘EMT-associated’ promoter to drive the Cre recombinase, as EMT is controlled and characterized by a plethora of transcriptional changes. In addition, it does not address the inherent plasticity and reversibility within the system: EMT does not represent an on-off switch but is a continuum with many cells expressing both epithelial and mesenchymal markers at a given time. Indeed, in a model of HER2-driven mammary cancer, early dissemination of tumor cells was associated with a partial EMT, wherein some epithelial cell properties were retained (Harper et al., 2016).

The use of intra-vital imaging has helped unravel the complexities associated with tumor cell plasticity and metastatic spread. In the MMTV-PyMT mammary tumor model, Beerling and colleagues used a fluorescent epithelial marker (E-cadherin-CFP) combined with intra-vital imaging to show that EMT is a reversible, plastic process and that mesenchymal cells that reach secondary sites can rapidly regain an epithelial phenotype (Beerling et al., 2016). In contrast with many other studies, it was possible to demonstrate this plasticity *in vivo* without experimental modulation of genes commonly thought to regulate EMT. Models that are able to accurately recapitulate the metastatic cascade without experimental gene modulation are key tools in uncovering the events involved, but in a way that reflects *in situ* processes as closely as possible. The use of lineage tracing to assess the contribution of EMT to metastasis has the potential to help uncover the gene signature of cells that are able to colonize secondary sites.

## Unraveling the polyclonal nature of metastasis

The increasing use of deep-sequencing analysis has helped elucidate the evolutionary history of metastatic lesions and has shed light on whether metastatic dissemination follows a linear or parallel model (Turajlic and Swanton, 2016). Evidence from a HER2-driven mouse mammary tumor model supports a parallel model of evolution and also highlights the importance of mouse models in demonstrating that early disseminated tumor cells are critical to the formation of metastatic lesions (Hosseini et al., 2016). Lineage tracing of CTCs and clusters of CTCs has also proven invaluable in the investigation of the origins of these clusters and single cells. By using a convertible double-fluorescent mammary tumor model, *ROSA^mT/mG^; MMTV-PyMT*, from which organoids (see Glossary, Box 1) were injected into the mammary fat pad of nonfluorescent hosts, Cheung et al. (2016) evidenced the polyclonal origin of a lesion in the lung, showing that it contained at least two separate clones based on the fluorescent reporters present. They found multicolored cell clusters at all stages of the metastatic cascade, including local disseminated and CTC clusters. The advent of Brainbow-2.1 (Livet et al., 2007) has allowed the detailed tracing of multiple cells by utilizing Cre recombinase and the stochastic expression of four fluorescent proteins from multiple copies of a single transgene, which can generate up to 90 distinguishable colors when multiple copies of Brainbow-2.1 are present per cell, due to the differential expression of each transgene (Fig. 3). By combining the transgene with Cre recombinase placed under tissue-specific promoters, it will be possible to more fully assess the clonal heterogeneity of a metastatic lesion using imaging alone (Fig. 3) or in combination with deep-sequencing techniques. One such example of this is Prorainbow, a novel fluorescently labeled mouse strain (*PB-Cre4; Pkd1^lox/lox^; Pten^lox/lox^;* *CMV-XFP/+*) that can be used to model prostate cancer (Fang et al., 2015). Although this model has not yet been used to look at the metastatic process, initial characterization indicates that it is an extremely promising advance in technology to allow assessment of metastatic colonization in many different mouse models.

## Arrest, extravasation and colonization at distant organs

Experimental metastasis assays have illustrated that multiple stromal components can be hijacked by tumor cells in the process of metastatic seeding, as detailed below. Platelets play an important role in facilitating metastatic dissemination. In addition to protecting cells in the bloodstream from natural killer (NK) cells, interaction of platelet integrins with collagen at specific regions of the endothelium may help tumor cell adherence at secondary organs and determine the site of metastatic tumor cell extravasation (Gay and Felding-Habermann, 2011; Ruggeri and Mendolicchio, 2007). A recent study identified that platelets promote tumor cell extravasation through adenosine triphosphate (ATP)-dependent activation of the endothelial P2Y2 receptor, which opens the vessel barrier to enable tumor cell extravasation and metastatic seeding (Schumacher et al., 2013). This was identified by utilizing both spontaneous and experimental metastasis models in syngeneic C57BL/6J mice. The utility of experimental metastasis models in studying these early events when tumor cells first reach the metastatic site was further highlighted by another group, who were able to show that metastatic seeding is promoted by coagulation pathways, in particular tissue factor (TF). By imaging myeloid-tumor cell interactions within the lung following intravenous injection of tumor cells, they demonstrated that TF-induced platelet clots attract recruitment of bone marrow-derived macrophages to support the survival of metastatic melanoma cells and inhibit NK cell-mediated destruction of micrometastases in the lung (Gil-Bernabe et al., 2012).

Using both spontaneous and experimental lung metastasis models of breast cancer, a distinct population of metastasis-associated macrophages (MAMs) has been characterized in the target organ (lung). Depletion of these MAMs using transgenic CD11b-diphtheria toxin receptor (DTR) mice significantly reduces metastatic seeding and persistent growth of breast cancer cells (Qian et al., 2011). One caveat of this approach is that administration of diphtheria toxin to CD11b-DTR mice selectively kills CD11b-expressing monocytes as well as macrophages (Stoneman et al., 2007). However, *ex vivo* intact lung imaging in the same model revealed that macrophages (and not monocytes) directly contact the extravasating tumor cells, and depletion of these macrophages significantly reduces the number of tumor cells that complete extravasation (Qian et al., 2009).

Adhesion signaling also plays an important role in the survival of tumor cells within the metastatic niche. Using an innovative method of experimental bone metastasis in which breast cancer cells were injected into the iliac artery of mice (both allograft and xenograft models), Wang et al. (2015) showed that bone seeding of multiple human and murine breast cancer cells is dependent on heterotypic adherens junctions between cancer cells and osteogenic cells. Thus, a number of models and experimental approaches have demonstrated the importance of the interaction of tumor cells with stromal cells in the metastatic niche that is required to support the outgrowth of secondary tumors.

## Dormancy in the metastatic niche

Metastases can arise from DTCs many years after the initial treatment and/or surgical removal of the primary tumor. This is because at the time of diagnosis, metastatic spread has already occurred but the resulting DTCs have entered a state of quiescence (Sosa et al., 2014). These metastases may be resistant to current therapies that are directed at proliferating cells; therefore, targeting dormant DTCs or preventing their reactivation from dormancy may be of clinical benefit. The factors that control tumor dormancy are poorly understood, but using a spontaneous model of breast cancer metastasis, DTCs were found to reside on the microvasculature of different organs and subsequent *in vitro* experiments demonstrated that distinct endothelial niches can induce dormancy through the secretion of thrombospondin-1 (Ghajar et al., 2013). A gain-of-function screen in an allograft model of mouse mammary carcinoma identified the bone morphogenetic protein (BMP) inhibitor Coco as a mediator of DTC reactivation (Gao et al., 2012). Coco stimulated the proliferation of DTC in the lungs through induction of a discrete gene expression signature that was associated with relapse to the lung but not to other organs where BMP is not active. This highlights the organ specificity of signals that control reactivation of DTCs in the metastatic niche. Bragado et al. (2013) provided further support to this by tracing spontaneous DTCs following subcutaneous transplantation of a head and neck squamous cell carcinoma cell line. They showed that transforming growth factor beta 2 (TGFβ2) signaling in the bone marrow initiates tumor dormancy, while the low levels of TGFβ2 signaling found in the lungs prevented long-term dormancy, resulting in outgrowth of metastatic lesions (Bragado et al., 2013). These studies support the concept of dormancy-permissive and -restrictive microenvironments that determine whether DTCs divide or remain quiescent. The collection and analysis of CTCs and DTCs from patients at different stages of the metastatic journey will provide important information and clinical validation of the functional regulators linked to the emergence of overt metastatic disease. This should provide an iterative framework for the further refinement of mouse models.

## Therapeutics and translation

Although mouse models have been invaluable in enhancing our understanding of the biology that drives the metastatic cascade, their utility as preclinical drug development tools is less well defined. The approval rate of oncology drugs remains poor, with recent research indicating that only 7.5% of oncology drugs that entered phase I clinical development, and 33.2% of drugs that entered phase III trials, were eventually approved (Toniatti et al., 2014). This highlights the need for more predictive and improved preclinical mouse models. The majority of preclinical studies that support clinical evaluation rely on the use of established cell lines grown ectopically in immune-deficient mice. These cell lines show limited tumor heterogeneity, and combined with the lack of appropriate human stroma and an intact immune system (Table 1), this contributes to the poor clinical predictivity of these models (Singh and Ferrara, 2012; Toniatti et al., 2014). In most studies, regression of primary tumor growth is used as an endpoint and no consideration is given to effects on metastatic disease (in most cases metastatic disease is not even modeled). By contrast, the majority of early clinical trials will involve patients with advanced metastatic disease and as the genetic and epigenetic landscape of metastases differ from the primary tumor, which is reflected in the response to treatment, identifying the most effective way to model this will have an impact on drug development programs. The ability to model this in preclinical models is challenging, but the resistance of metastatic disease to current therapies and the realization that >90% of cancer-related mortality is due to metastatic disease progression highlights the need for new approaches to be considered. In addition to developing improved models, a better understanding of how best to implement currently available models could provide benefits (Francia et al., 2011; Steeg, 2016). For example, experimental metastasis models have been used widely, but what needs to be considered is the cell population that is being targeted in these models. Do they reflect the outgrowth of latent tumor cells or the subsequent growth of macrometastatic lesions? Such nuances are currently difficult to address in most models and are not often considered when carrying out drug intervention studies.

Despite the limitations associated with the use of established cell lines in immune-deficient animals, they can provide important insights into the potential clinical activity of drugs, for which differential responses to therapies have been reported between primary and metastatic lesions, thus reflecting what is seen in the clinic (Francia et al., 2011). Another important consideration is how closely we can recapitulate clinically relevant intervention strategies. Surgical removal of the primary tumor followed by adjuvant treatment to prevent recurrence is widely used in clinical practice. Spontaneous metastasis models that utilize cell lines (xenograft, allograft) are particularly amenable to modeling adjuvant treatments, although they are limited to a few models where the primary tumor can be easily resected (e.g. Cruz-Munoz et al., 2008; Ebos et al., 2009). Although these experiments can be technically challenging and time-consuming, they have provided useful insight into the activity of drugs in the clinical setting. For example, studies with transplantable models have helped to unravel the effects of anti-angiogenic therapies in the adjuvant setting (Kerbel et al., 2013) and shed light on the possible mechanisms responsible for the disappointing results of recent Phase III clinical trials in metastatic breast cancer (Bridgeman et al., 2017).

The benefits of GEMMs in generating tumors that develop in the organ of origin (autochthonous) in immune competent hosts has led to their adoption for preclinical assessment of drug response and mechanisms of resistance (Gopinathan et al., 2015; van Miltenburg and Jonkers, 2012). GEMMs are very useful for studying early and late stages of disease, and studies showing the clinical predictivity of lung and pancreatic cancer GEMMs to chemotherapy are encouraging (Singh et al., 2010), but this needs to be more widely validated in other models and the impact on metastatic disease evaluated. These models are often limited by growth of the primary tumor, which necessitates cull of the animals and thus true effects on metastasis-associated survival cannot be monitored (Karim et al., 2013). Most GEMMs are not amenable to resection of the primary tumor and thus dissecting effects on primary tumor growth from specific effects on metastatic disease are complicated, as neoadjuvant and adjuvant studies cannot be carried out although excision of primary pancreatic tumors in the KPC model is being trialed (Gopinathan et al., 2015). In addition, in the context of mammary tumors, the development of transplantation models in which fragments of tumors are re-implanted into recipient mice allows resection of the primary tumor and the subsequent monitoring of metastatic spread. This reduces the latency and variability in metastatic dissemination making this approach more amenable to drug studies. This opens up the possibility of using these models for testing adjuvant therapies and importantly for assessing the potential of new immunotherapies (Coffelt et al., 2015).

PDX models are also predicted to be a major advance in preclinical testing platforms as they recapitulate the tumor heterogeneity that plays such an important role in tumor biology, including response to therapy. A number of therapeutic studies have demonstrated their value in linking response with genetic alterations and identifying mechanisms of resistance and biomarkers, while use of humanized mice recipients will further enhance their value as we look at the potential for testing immunotherapies (Byrne et al., 2017). However, the cost and length of time required to conduct studies in PDX models is restrictive and, as yet, their utility in assessing effects on metastatic spread is not clear. Although spontaneous metastases do develop, this is limited to a relatively small number of PDX models, with orthotopic injection of tumor fragments being more successful in modeling metastatic spread (Pompili et al., 2016). Moreover, the asynchronous development of metastatic disease in these models would require large cohorts of animals, which further increases the cost of such studies. Generation of PDX from primary tumors and metastatic sites from the same patient that can be transplanted orthotopically would allow direct evaluation of drugs in the metastatic setting.

The increasing use of imaging modalities that allow noninvasive longitudinal monitoring of metastases will help more accurate detection and quantification of metastatic disease in deep tissue sites. This technology is thus likely to enhance the usability of GEMMs and PDXs. Recent advances in preclinical magnetic resonance imaging (MRI), computed tomography (CT), positron emission tomography (PET) and single-photon emission computed tomography (SPECT) are showing promise (Fleten et al., 2017; Marien et al., 2017; Sanches et al., 2015; Taromi et al., 2016).

A greater understanding of the pathways that drive metastatic spread and what is achievable in the clinical setting gives hope that anti-metastatic therapies will be viable in the future. For example, targeting metastatic colonization, which is often the rate-limiting step in the metastatic cascade holds great potential, but it will be important to provide robust preclinical evidence of activity in appropriate animal models that accurately reflect the clinical scenario in which the therapy will ultimately be tested in. Careful thought to clinical trial design with appropriate primary endpoints is also essential.

## Future outlook

Mouse models have been essential in advancing our understanding of the biological processes that drive tumor progression. However, metastatic cancer remains the main cause of cancer-related deaths, with new treatments desperately needed. Increasing our understanding of what drives metastatic spread is key to achieving this goal. This will require the further development and refinement of mouse models to more faithfully mimic human disease and progression.

A number of technological advances have allowed the further development of GEMMs. These include combined use of the Cre-*loxP* and Flp-FRT systems (Schönhuber et al., 2014) that allow sequential activation or inactivation of genes, thereby enabling human disease progression to be mimicked more closely, which may provide more representative metastatic progression models. This also opens up the possibility of targeting both tumor and stromal cells in the same model, which is an important consideration when studying metastatic progression. In addition, the CRISPR/Cas9 gene editing system (see Glossary, Box 1) has been used to successfully introduce targeted mutations in GEMMs, enabling more rapid validation and characterization of putative cancer genes that are being uncovered by the large-scale sequencing efforts currently underway using human tissue samples (Annunziato et al., 2016; Chiou et al., 2015; Sánchez-Rivera et al., 2014; Weber et al., 2015). It is hoped that this will help in the design of better GEMMs that more readily recapitulate the metastatic tropism seen in human disease. The development of humanized mouse models that incorporate human-derived stromal components, including CAFs and immune cell populations, will help with regards to metastasis to particular sites (Shultz et al., 2012). For example, human bone discs and engineered human bone environments have been used to model bone metastases (Holen et al., 2015; Wright et al., 2016). RNA interference and CRISPR/Cas9 screens have been used to identify metastatic drivers in *in vivo* transplantation models (Chen et al., 2015; Murugaesu et al., 2014); the validation of these and improvements in such screening approaches will help in the development of future GEMMs.

The design of more biologically relevant *in vitro* models that try to recapitulate the complexity of the tumor microenvironment is a major area of ongoing research. Models include co-culture of tumor cells with different stromal cell types and 3D engineered matrixes, with developments in 3D bioprinting and microfluidics platforms having the potential to significantly impact on the design and reconstruction of the complex tumor microenvironment (Albritton and Miller, 2017). Coupled with the use of high-resolution real-time imaging, microfluidics devices have been used to study different components of the metastatic cascade such as intra- and extravasation (Jeon et al., 2015; Zervantonakis et al., 2012). The design of these models could be geared towards addressing specific questions about the biology that drives metastatic behavior. Importantly, such *in vitro* models have the potential to be used as drug screening platforms and could guide the preclinical testing of new agents in mouse models.

Advances in intra-vital imaging have shown great potential for elucidation of the close interplay between tumor and host cells (Cuccarese et al., 2017; Hawkins et al., 2016; Headley et al., 2016; Lee et al., 2015; Szulczewski et al., 2016), and fundamental aspects of the metastatic process. For example, use of a Cre recombinase-driven reporter system has allowed the visualization of extracellular vesicle exchange between tumor cells and has provided further insight into the biological consequences and potential impact on metastatic potential (Zomer et al., 2015). The use of optical windows to allow imaging in the metastatic niche provides both spatial and temporal information on the behavior of cancer cells; another major advance (Entenberg et al., 2015; Headley et al., 2016; Ritsma et al., 2012; Rodriguez-Tirado et al., 2016). The ability of such imaging approaches to shed light on the localization and activity of drugs within the tumor microenvironment (Dubach et al., 2016; Junankar et al., 2015; Tipping et al., 2016) also provides a more sophisticated platform for evaluation of new therapies.

Although much is known about the biological pathways that control the individual steps in the metastatic cascade, a number of important questions remain. Uncovering the underlying mechanisms that govern the enormous diversity in the onset and target organs affected in patients and identifying whether there are common mechanisms at play will be important. In addition, understanding the fundamental differences between primary and secondary tumors and the drivers of metastatic colonization is essential to identifying strategies for targeting metastatic disease, and would have a major impact on the survival of cancer patients. The technological advances in the generation of mouse models that better mimic human disease, combined with advances in imaging, will allow for the translation of such research into meaningful treatments.
